# The effects of different shapes of capsulorrhexis on postoperative refractive outcomes and the effective position of the intraocular lens in cataract surgery

**DOI:** 10.1186/s12886-019-1068-3

**Published:** 2019-02-21

**Authors:** Shixu Li, Yiping Hu, Ran Guo, Yushuang Shao, Jiangyue Zhao, Jinsong Zhang, Jing Wang

**Affiliations:** Department of Ophthalmology, the Fourth Affiliated Hospital of China Medical University, Eye Hospital of China Medical University, the Key Lenticular Laboratory of Liaoning Province, Shenyang, 110005 China

**Keywords:** Effective intraocular lens position, Capsulorrhexis, Postoperative refractive outcomes

## Abstract

**Background:**

To evaluate the effects of anterior capsular opening size on deviation from predicted refraction and the effective position of the intraocular lens (ELP) in cataract surgery.

**Methods:**

Nonrandomized clinical trial. Eighty patients (80 eyes) with simple age-related cataracts were treated from May 2018 to September 2018 at the Fourth Affiliated Hospital of China Medical University. All patients undergoing phacoemulsification received intraocular lens based on the voluntary principle. Forty eyes were implanted with the C-loop haptic intraocular lens (AMO Tecnis ZCB00) while the other 40 eyes were implanted with the plate haptic intraocular lens (CT ASPHINA 509 M). Follow-up visits were conducted postoperatively at 1 week, 1 month, and 3 months during which patients underwent refraction and data collection after pupil dilation, which included anterior segment photography and Scheimpflug imaging by Pentacam. The area, horizontal and vertical diameter of the capsulorrhexis, circularity, decentration, and package were analysed using the image analysis software Image-Pro-Plus 6.0,then evaluated the relationship between the different shapes of capsulorrhexis with deviation from predicted refraction and ELP in cataract surgery.

**Results:**

Deviation from predicted refraction and all of the parameters of capsulorrhexis were not correlative in the 509 M IOL group, however, in the Tecnis IOL group, while the deviation from predicted refraction and all of the capsulorrhexis parameters were not correlative at 1 week, the deviation from predicted refraction did correlate with capsulorrhexis area, horizontal diameter at 1 month (*P* = 0.029, *P* = 0.048), and with capsulorrhexis area, vertical diameter at 3 months (*P* = 0.03, *P* = 0.017). The ELP correlated with package in both groups postoperatively (r > 0, *P* < 0.05), but there is no other capsulorrhexis parameters correlated with ELP in the 509 M IOL group (all *P* > 0.05). For the Tecnis IOL group, the ELP and capsulorrhexis area were correlated at 1 week and 1 month, while the ELP and horizontal diameter, the ELP and vertical diameter were correlated at 1 week, but did not correlate with the other capsulorrhexis parameters in the Tecnis IOL group (all *P* > 0.05).

**Conclusions:**

The shape of the capsulorrhexis has an effect on postoperative refractive outcomes and the effective position of the intraocular lens in cataract surgery, and plate haptic intraocular lenses have better refractive stability than C-loop haptic intraocular lenses.

**Trial registration:**

ChiCTR1800015638,2018-04-12.

## Background

Currently, phacoemulsification and intraocular lens implantation are the most effective treatments for cataract. With improvements in surgical techniques and the development of the refractive field, patients have increasingly high precision requirements for long-term and stable optical quality following an operation, and this has become a common pursuit for both doctors and patients. Continuous circular capsulorrhexis (CCC) is a common method in cataract surgery. This method is broadly popular and has an irreplaceable special status in the cataract field. This method allows the capsule to remain relatively intact to ensure accurate implantation of the intraocular lens, effectively preventing the optical centre of the intraocular lens from moving or tilting. A capsule that is irregularly shaped, eccentric, or that does not fully cover the optic portion of the intraocular lens may lose these advantages and may lead to the postoperative refraction that does not match the preoperative predicted refraction, which will ultimately reduce the patient’s visual quality and influence the outcome of cataract surgery.

The effective intraocular lens position is defined as the vertical distance from the posterior corneal apex to the optical plane of the intraocular lens on the visual axis. It reflects the longitudinal position of the intraocular lens in the eye, and the fusion and fibrosis processes of the lens capsule produce the forward and backward forces, while the ELP reflects the unbalanced result of those forces [1]. When the intraocular lens moves forward it causes a myopic shift, and when it moves backward it causes a hyperopic shift, therefore, the ELP determines the refractive condition after cataract surgery [2]. This paper intends to explore the effects of the morphological parameters of the capsulorrhexis on postoperative refractive outcomes and the effective position of the intraocular lens.

## Methods

### Patients selection

This study was approved by the Fourth Affiliated Hospital of China Medical University. All research and data collection practices adhered to the tenets of the Declaration of Helsinki and good clinical practices, and all patients provided a signed informed consent to participate in a clinical research study.

Eighty patients (80 eyes) with simple age-related cataracts were treated from May 2018 to September 2018 in the Fourth Affiliated Hospital of China Medical University. We specifically included patients who were bodily healthy and underwent a successful implantation of an IOL. The exclusion criteria for this study were irregular astigmatism, corneal opacity, glaucoma, retinal disease, a history of ocular inflammation, a history of ocular trauma, any other previous intraocular surgery, and intraoperative complications including anterior or posterior capsular tears or postoperative macular edema. The patients were divided into 2 groups according to the different intraocular lenses implanted: 40 eyes were implanted with the C-loop haptic intraocular lens (AMO Tecnis ZCB00) while the other 40 eyes were implanted with plate haptic intraocular lenses (CT ASPHINA 509 M).

### Surgery

All phacoemulsification and IOL implantations were performed by one experienced surgeon under topical anaesthesia with 0.5% proparacaine hydrochloride. The complete anterior capsular opening was made approximately 5.5 mm in diameter using capsulorrhexis forceps. After phacoemulsification of the nucleus and aspiration of the cortex, the Tecnis IOL or 509 M IOL was injected into the capsular bag, and the IOL centration was confirmed again after viscoelastic removal. The Haigis formula was used for the lens power selection. All patients received an anti-inflammatory treatment consisting of Tobramycin and Dexamethasone eye drops (5 ml (15 mg/5 mg)) (TobraDex®, Alcon) that were used every 6 h for 2 months along with 0.1% Bromfenac Sodium Hydrate eye drops (BRONUCK®, SENJU) that were used every 12 h for 1 month.

### Postoperative evaluation

**Fig. 1:**
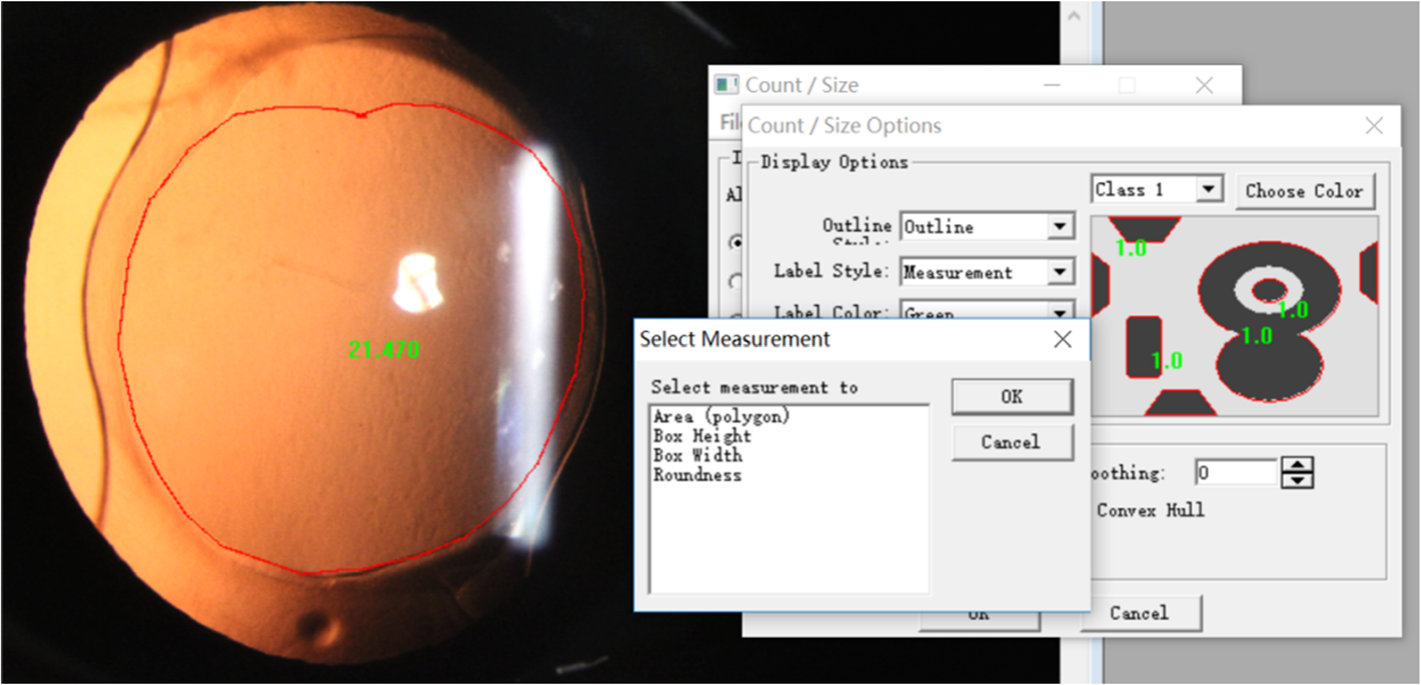
Using Image-Pro-Plus 6.0 to analyse the capsulorrhexis parameters including the capsulorrhexis area, horizontal diameter, vertical diameter, circularity, decentration, and package

Postoperative examinations were performed at 1 week, 1 month, and 3 months after the surgery. All patients were measured for subjective refraction that was converted into spherical equivalent (SE), and the deviation from predicted refraction was the difference between postoperative SE of the subjective refraction and the predicted refraction. The patients then underwent slit-lamp digital photography measurements and Pentacam measurements, and the capsulorrhexis were documented using digital retroillumination photographs through dilated pupils. All photographs were imported into Image-Pro-Plus 6.0 to analyse the capsulorrhexis parameters including the capsulorrhexis area, horizontal diameter, vertical diameter, circularity, decentration, and package (Fig. 1). Circularity is defined by the formula: Circularity = 4π × area/perimeter^2^, an index of 1.0 denotes a theoretically perfect circle, and the lower the index, the more irregular the capsulorrhexis [3]. Decentration is the vector between the centre of the pupil and the centre of the capsulorrhexis outline. Package is expressed as the ratio of the minimum distance to the maximum distance of the capsule edge from the optical plane edge (package = minimum distance/maximum distance).

### Statistical analyses

Statistical analysis was performed using SPSS 24.0 for Windows (SPSS Inc., Chicago, IL, USA). The normality of all data distributions was confirmed using the Kolmogorov-Smirnov test. Paired t-tests were used to compare normally distributed data, while nonparametric data were analysed by a one-way ANOVA. Spearman correlation tests were used to determine the correlations between capsulorrhexis parameters and postoperative refractive outcomes or correlations between capsulorrhexis parameters and ELP. Statistical significance was defined as a *P* value that was less than 0.05.

## Results

### Patient characteristics

Our study protocol included 80 eyes from 80 patients who were aged between 62 and 74 years. Patient recruitment took place from May 2018 to September 2018: Forty eyes were implanted with the C-loop haptic intraocular lens (AMO Tecnis ZCB00) and the other forty eyes were implanted with plate haptic intraocular lenses (CT ASPHINA 509 M). The patient demographics and IOL models for the two groups are summarized in Table 1. There was no significant (*P* > 0.05) difference between the two groups (Table 1).

**Table 1:** Demographic and clinical information for patients included in this study

Groups	Age(years)	Eyes(n)	Sex		Laterality	
			Male	Female	Right	Left
509 M	62.54 ± 11.81	40	24	16	20	20
Tecnis	63.00 ± 9.08	40	18	23	21	19
t	0.376		0.841		2.875	
*P* value	0.71		0.365		0.098	

### Capsulorrhexis morphological features

The capsulorrhexis area, horizontal diameter, vertical diameter, circularity, decentration, and package were not significantly different in the 509 M IOL group at 1 week, 1 month, or 3 months after the operation (all *P* > 0.05). However, the capsulorrhexis area, particularly the horizontal diameter, was significantly different in the Tecnis IOL group at 1 week to 1 month, and at 1 week to 3 months (all *P* < 0.05) (Table 2).

**Table 2:** The two groups’ difference value on capsulorrhexis morphological features at different time points

Groups		1 w&1 m	1 w&3 m	1w&3 m	1 w&1 m	1w&3 m	1 m&3 m	1 w&1 m	1 w&3 m	1 m&3 m
		Area(mm2)			Horizontal diameter(mm)			Vertical diameter(mm)		
509 M	D-value	0.21 ± 2.72	0.019 ± 2.98	0.38 ± 0.98	0.008 ± 0.4	−0.12 ± 0.42	0.42 ± 0.29	0.04 ± 0.43	−0.05 ± 0.55	−0.03 ± 0.31
	t	0.562	0.04	2.354	0.146	−0.184	0.866	0.719	−0.578	− 0.532
	P	0.577	0.968	0.024	0.884	0.855	0.392	0.475	0.567	0.598
Tecnis	D-value	1.02 ± 1.06	1.09 ± 1.30	0.07 ± 0.87	0.15 ± 0.18	0.15 ± 0.27	−0.001 ± 0.19	0.07 ± 0.21	0.06 ± 0.19	−0.01 ± 0.11
	t	3.816	3.439	0.085	2.692	2.374	−0.332	1.903	1.309	−0.771
	P	0.001^*^	0.003^*^	0.933	0.012^*^	0.030^*^	0.744	0.067	0.209	0.451
		Circularity			Decentration(mm)			Package		
509 M	D-value	0.01 ± 0.11	0.005 ± 0.05	−0.01 ± 0.13	0.02 ± 0.16	0.03 ± 0.19	0.03 ± 0.13	−0.005 ± 0.81	−0.17 ± 0.08	0.01 ± 0.70
	t	0.812	0.621	−0.516	0.827	1.085	1.502	−1.295	−1.214	0.589
	P	0.42	0.538	0.609	0.412	0.285	0.142	0.201	0.232	0.559
Tecnis	D-value	−0.16 ± 0.05	0.01 ± 0.06	0.03 ± 0.03	−0.07 ± 0.13	−0.03 ± 0.13	0.04 ± 0.11	−0.03 ± 0.12	− 0.003 ± 0.07	0.02 ± 0.15
	t	−1.37	0.591	3.348	−1.709	−0.81	1.099	−1.135	−0.174	−1.536
	P	0.182	0.563	0.004^*^	0.099	0.43	0.287	0.266	−0.174	0.143

^*^P < 0.05

### Morphological features of Capsulorrhexis

One-way ANOVA was performed in the 509 M IOL group and the Tecnis IOL group and the results showed that the Tecnis IOL group had a significantly higher degree of package than the 509 M IOL group at 1 month (*P* < 0.001), but there were no other differences in the morphological features of capsulorrhexis (all *P* > 0.05) (Table 3).

**Table 3:** Comparison of the capsulorrhexis parameters in the 509 M IOL group and the Tecnis IOL group at 1 week, 1 month, and 3 months postoperatively

		Area (mm2)	Horizontal diameter (mm)	Vertical diameter (mm)	Circularity	Decentration (mm)	Package
1 w	509 M	21.96 ± 4.76	5.39 ± .55	5.19 ± .63	.85 ± .060	0.34 ± .18	.072 ± .12
	Tecnis	21.15 ± 3.39	5.23 ± .40	5.17 ± .49	0.84 ± .050	0.28 ± .16	0.11 ± .14
	F	0.673	1.951	0.043	0.034	2.221	1.544
	P	0.414	0.166	0.836	0.854	0.140	0.218
1 m	509 M	21.74 ± 4.68	5.89 ± .59	5.15 ± .71	.84 ± .12	.32 ± .18	.087 ± .12
	Tecnis	20.31 ± 3.17	5.12 ± .35	5.11 ± .48	.86 ± .03	0.32 ± .20	0.14 ± .17
	F	2.774	3.615	0.363	0.186	0.036	97.533
	P	0.101	0.062	0.549	0.668	0.85	0.000 ^*^
3 m	509 M	21.46 ± 4.79	5.34 ± .58	5.18 ± .82	0.84 ± .05	0.30 ± .19	.10 ± .15
	Tecnis	20.17 ± 3.23	5.10 ± .44	5.12 ± .44	0.84 ± .03	0.34 ± .16	0.12 ± .13
	F	0.259	1.118	0.020	0.200	1.297	0.004
	P	0.613	0.295	0.887	0.657	0.259	0.951

### Postoperative refractive outcomes

**Fig. 2:**
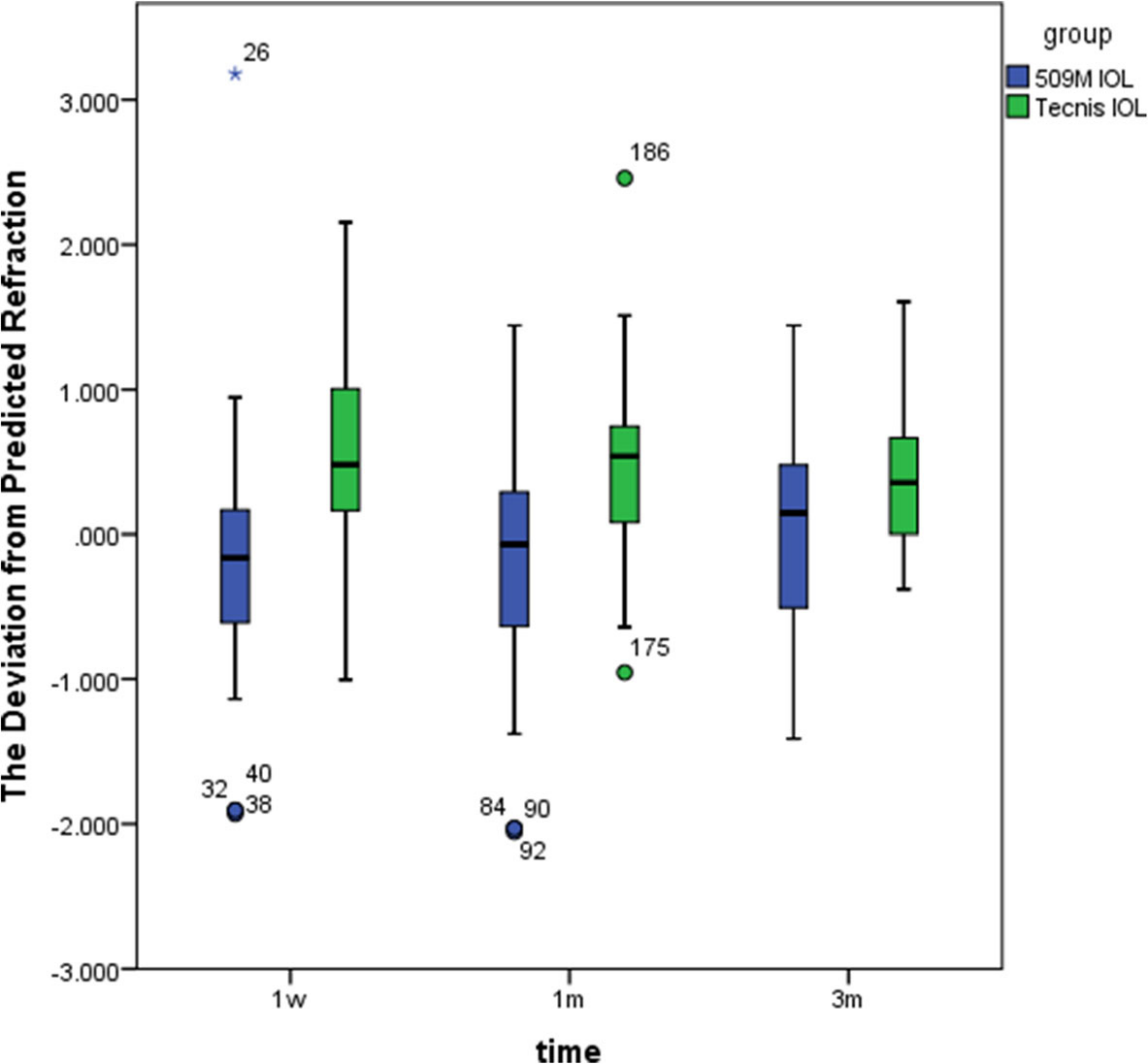
The deviation from predicted refraction of the 509 M IOL group and the Tecnis IOL group at 1 week, 1 month, and 3 months postoperatively

There was no significant difference in postoperative refractive outcomes between two groups at 1 week, 1 month, and 3 months. (509 M group: t = − 0.891 *P* = 0.381, Tecnis group: t = 0.325 *P* = 0.750, *P* all> 0.05). This finding indicates that the postoperative dioptre is stable across 3 months and does not show differences over time (Fig. 2).

### Effective position of the intraocular Lens

**Fig. 3:**
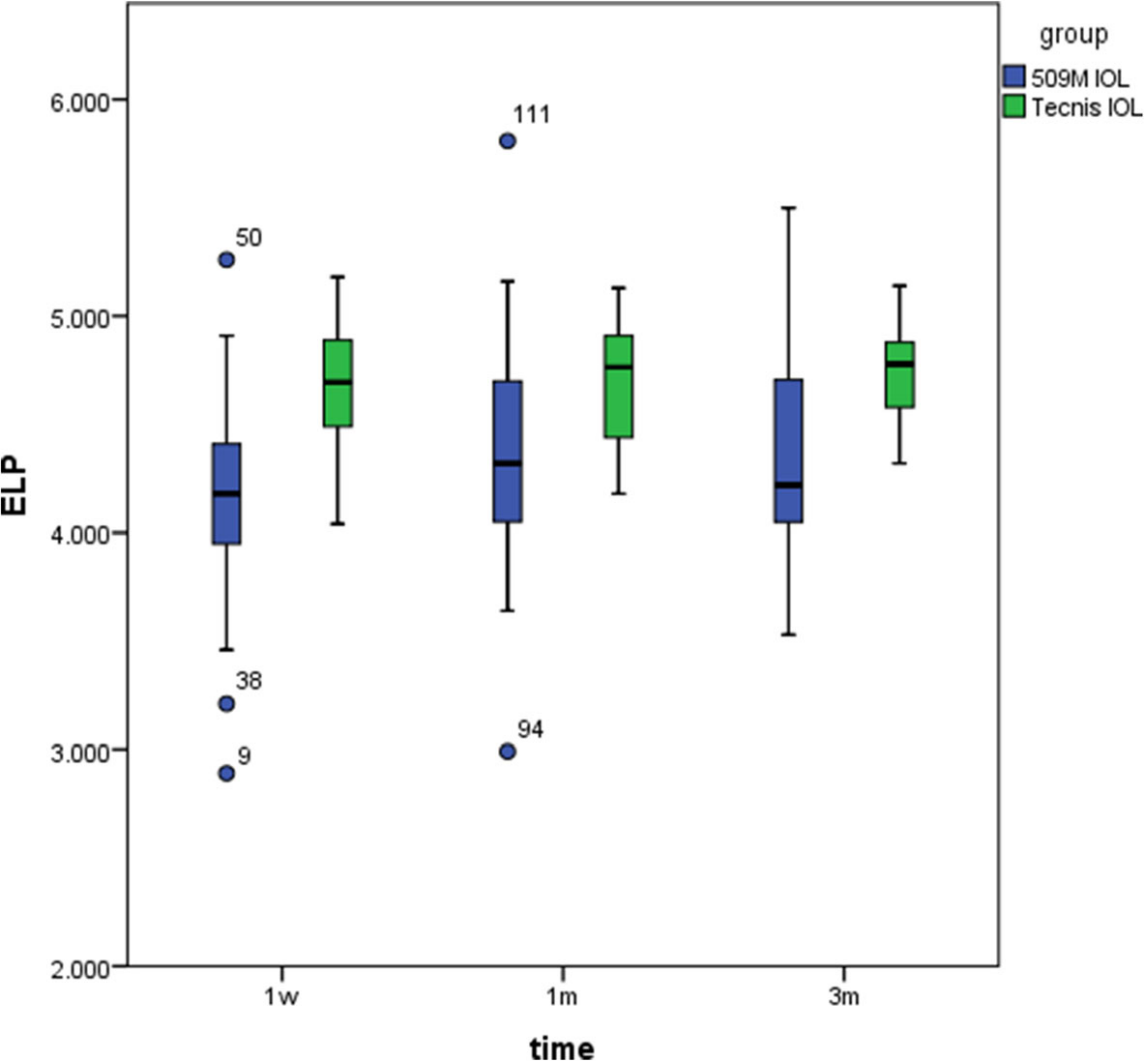
The ELP of the 509 M IOL group and the Tecnis IOL group at 1 week, 1 month, and 3 months postoperatively

A non-parametric test for the effective position of the intraocular lens indicated that the effective positions of the intraocular lens in the 509 M IOL group at 1 month and 3 months were significantly larger than that of postoperative 1 week (*P* = 0.023, *P* = 0.038); however, since the ELP tends towards stability after 1 month, there was no significant difference between 1 month and 3 months (*P* = 0.961). For the Tecnis IOL group, no statistical differences were found at 1 week, 1 month, or 3 months after the operation (all *P* > 0.05) (Fig. 3).

### The effect of Capsulorrhexis morphological features on the deviation from predicted refraction

**Fig. 4:**
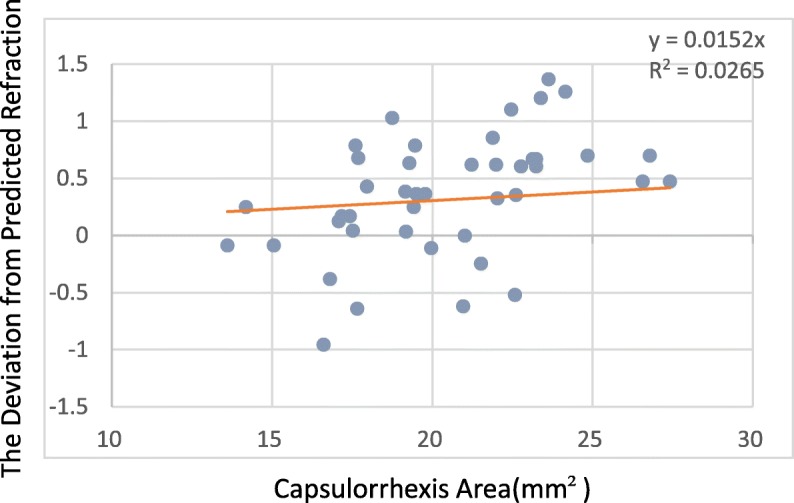
For every 1-mm2 increase in area, the deviation from predicted refraction increases by 0.0152 D

**Fig. 5:**
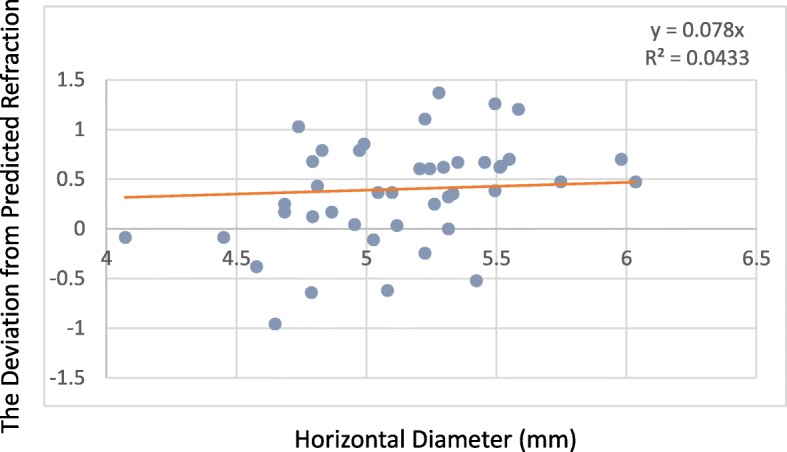
For every 1-mm increase in the horizontal diameter, the deviation from predicted refraction increases by 0.07 D

**Fig. 6:**
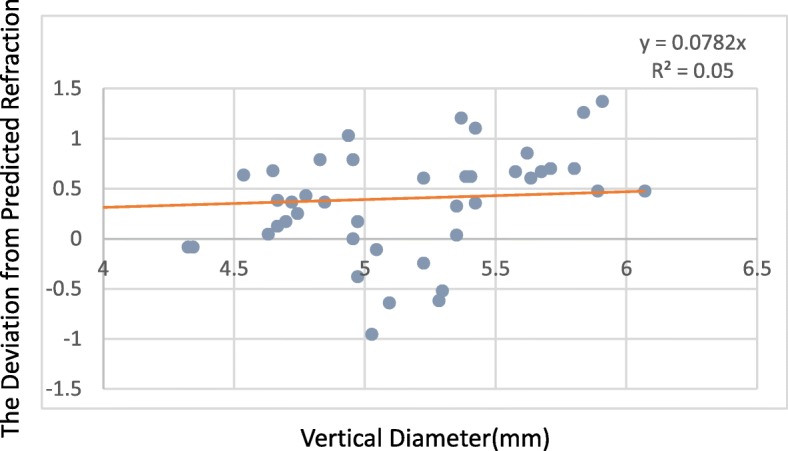
For every 1-mm increase in the vertical diameter, the deviation from predicted refraction increases by 0.05 D

The deviation from predicted refraction and all capsulorrhexis parameters were not correlated in the 509 M IOL group. However, for the Tecnis IOL group, while the deviation from predicted refraction and all capsulorrhexis parameters were not correlated at 1 week, the deviation from predicted refraction did correlate with the capsulorrhexis area and horizontal diameter at 1 month (*P* = 0.029, *P* = 0.048),and the capsulorrhexis area and vertical diameter at 3 months (*P* = 0.03, *P* = 0.017) (Table 4). The deviation from predicted refraction showed positive correlations with the change in the capsulorrhexis area, the horizontal diameter, and the vertical diameter (Figs. 4, 5 and 6). However, for the 509 M IOL, the morphological features of the capsulorrhexis showed no correlation with the deviation from the predicted refraction (all *P* > 0.05).

**Table 4:** Correlation of the morphological features of capsulorrhexis and the deviation

		Area(mm^2^)			Horizontal diameter(mm)			Vertical diameter(mm)			Circularity			Decentration(mm)			Package			ELP		
		1 w	1 m	3 m	1 w	1 m	3 m	1 w	1 m	3 m	1 w	1 m	3 m	1 w	1 m	3 m	1 w	1 m	3 m	1 w	1 m	3 m
509 M	r	0.06	−0.01	−0.22	0.04	−0.04	−0.15	0.01	−0.08	−0.19	−0.09	0.05	0.10	−0.02	−0.05	−0.05	−0.07	−0.05	0.07	0.09	0.12	0.07
	P	0.68	0.96	0.21	0.76	0.80	0.40	0.99	0.61	0.26	0.52	0.70	0.57	0.88	0.75	0.79	0.65	0.73	0.71	0.54	0.43	0.69
Tecnis	r	0.19	0.41	0.53	0	0.52	0.46	0.36	0.33	0.57	−0.25	−0.14	0.42	−0.15	0.13	0.25	−0.12	−0.09	−0.24	0.50	0.45	0.29
	P	0.33	0.03^*^	0.03^*^	0.99	0.05^*^	0.06	0.06	0.09	0.02^*^	0.19	0.48	0.09	0.49	0.50	0.34	0.54	0.65	0.36	0.007^*^	0.02^*^	0.32

^*^P < 0.05

### The effect of Capsulorrhexis morphological features on the ELP

The ELP correlated with package in both groups postoperatively (r > 0, *P* < 0.05), but there is no other capsulorrhexis parameters correlated with ELP in the 509 M IOL group (all *P* > 0.05). For the Tecnis IOL group, the ELP and capsulorrhexis area were correlated at 1 week and 1 month, while the ELP and horizontal diameter, the ELP and vertical diameter were correlated at 1 week, but did not correlate with the other capsulorrhexis parameters in the Tecnis IOL group (all *P* > 0.05) (Table 5).

**Table 5:** Correlations of the morphological features of capsulorrhexis and the ELP

		Area(mm^2^)			Horizontal diameter(mm)			Vertical diameter(mm)			Circularity			Decentration(mm)			Package		
		1 w	1 m	3 m	1 w	1 m	3 m	1 w	1 m	3 m	1 w	1 m	3 m	1 w	1 m	3 m	1 w	1 m	3 m
509 M	r	0.19	0.11	−0.04	0.01	−0.07	−0.09	0.16	0.11	0.02	0.24	0.08	−0.07	−0.26	− 0.22	−0.27	0.25	0.30	0.36
	P	0.17	0.43	0.83	0.99	0.64	0.61	0.24	0.45	0.9	0.08	0.59	0.67	0.06	0.12	0.10	0.067	0.03^*^	0.03^*^
Tecnis	r	0.58	0.437	0.39	0.48	0.34	0.29	0.54	0.36	0.44	0.02	0.11	−0.27	−0.03	0.23	0.51	−0.41	−0.30	− 0.54
	P	0.001^*^	0.02^*^	0.15	0.008^*^	0.07	0.29	0.003^*^	0.05	0.10	0.92	0.58	0.34	0.89	0.22	0.05	0.03^*^	0.11	0.04^*^

^*^P < 0.05

## Discussion

Currently, with the development of refractive cataract technology, the requirement for accurate postoperative refraction is increasingly high, and surgeons are required to design the best operation plan perioperatively. There are many factors that affect the refractive outcomes of cataract surgery, including the influence of small surgical incisions, functional intraocular lens, advanced instruments and the surgical navigation system, these factors have been gradually solved, but irregular anterior capsular openings also affect the refractive outcomes, especially the size and shape of the capsular bag and the effective intraocular lens position [4, 5]. Therefore, we designed a prospective cohort study to investigate the relationships between the morphological parameters of capsulorrhexis and deviation from predicted refraction and the effective position of the intraocular lens.

Two types of intraocular lenses were included in this study. The Tecnis ZCB00 IOL is designed by C-loop haptic and the 509 M IOL is designed by plate haptic. The Tecnis IOL material is hydrophobic acrylate, designed by OptiEdge edge. This design reduces the incidence of posterior capsule opacification and improves the stability of the postoperative intraocular lens. The 509 M IOL is a single-piece hydrophilic acrylic intraocular lens and is more compatible with human tissue, while the plate haptic design makes the intraocular lens more stable and centred. Additionally, the analysis showed that there was no significant difference in the total-eye predicted spherical aberration between the two groups (P > 0.05),therefore, we chose these two different designs of intraocular lens to represent the two commonly used aspherical monofocal IOLs, which are typical and representative. The morphological features of capsulorrhexis (including the area of capsulorrhexis, horizontal diameter, vertical diameter, circularity, decentration, and package) that were measured at 1 week, 1 month, and 3 months showed that the 509 M IOL does not have significant differences over time. However, the area of capsulorrhexis and the horizontal diameter of the Tecnis IOL showed significant differences at 1 week to 1 month and at 1 week to 3 months. These results show that the Tecnis IOL’s capsulorrhexis had the largest area and horizontal diameter at 1 week after the operation, and with the passage of time, the area and horizontal diameter gradually decreased up to 1 month and remained stable to 3 months. These findings indicate that the area and the horizontal diameter gradually stabilize at 1 month after the operation. Further, the circularity showed significant differences at 1 month to 3 months postoperatively. These differences compared to the 509 M IOL, are likely due to the design of the IOL loops. The contractive force around the C-loop haptic IOL is not equivalent on every side of the capsule bag, and the parts of the IOL loops that do not completely and tightly contact the capsule bag can result in poor stability. Therefore, in terms of stability, the C-loop haptic IOL is not as good as the 509 M IOL [6]. In addition, capsule contraction syndrome occurs at 1 month after surgery, which leads to decreases in the area and horizontal diameter of the capsulorrhexis. As a result, we believe that the stability of the intraocular lens with the plate haptic design is better than that of the C-loop intraocular lens, and they have lower requirements for capsulorrhexis. Meanwhile, some studies show that the material of the optical surface also affects the contraction of the anterior capsule.

The effective position of the intraocular lens can reflect the longitudinal position of the intraocular lens in the eye. It has been shown that changes in anterior chamber depth of approximately 720 μm lead to a 1.00 D refractive deviation [7]. Moving forward to the retina leads to a myopic deviation while moving backward to the retina leads to a hyperopic deviation. Therefore, the ELP is particularly important for cataract surgery, especially for patients with refractive intraocular lens implantation [8, 9].

The effective position of the intraocular lens is related to the fibre shrinkage, capsular opacity, and the material of lens [10, 11]. If the opening of the anterior capsular is too small, it can cause contraction of the capsular bag that leads to the displacement of the ELP. Therefore, when the size of the anterior capsular is only wrapped around the edge of the optic surface of the intraocular lens (5.5 mm in diameter), the contraction of the capsular bag can be avoided and the influence on the ELP can be minimized [12].

These results showed that the effective position of the intraocular lens increased gradually at 1 week, 1 month, and 3 months in the 509 M IOL group, but there was no significant difference of the ELP in the Tecnis IOL at 1 week, 1 month, and 3 months after the operation. There are reports that this difference is related to the mechanical properties of the IOL [13, 14]. Different IOL edges have different effects on the axial motion of the intraocular lens. This finding may be because the 509 M IOL is a plate haptic design, and the four points of the intraocular lens all move forward during the contraction of the anterior capsule. This movement can result in the central axial force of the IOL moving backward, which leads to an increase in the effective position of the intraocular lens. For the C-loop haptic IOL, the small gap between the IOL loops and the optical zone may reduce the influence on the ELP during from the contraction of the anterior capsule.

The effective position of the intraocular lens may also be related to the material and design of the intraocular lens, which can affect the incidence of posterior cataract. The hydrophobic acrylate material lens is designed with a square edge, which can better block the movement of lens epithelial cells in the equatorial region and reduce the occurrence of posterior cataract. The hydrophilic acrylate material lens provides a better matrix structure for cell migration, leading to an increase in the rate of posterior cataract [15]. The axial movement of the lens depends on the forward and backward forces generated during the contraction of the capsule. The postoperative increase of ELP in the 509 M group and the stability of the ELP in the Tecnis group showed that the axial stability of the hydrophobic acrylate material was relatively better.

A precisely positioned, predictably sized CCC not only is an important guarantee for the surgeon to complete the operation safely but also ensures a lasting curative effect for the patient after surgery. In this study, the effects of the capsulorrhexis morphological features on the deviation from predicted refraction and ELP were observed by using the relevant parameters of area, horizontal diameter, vertical diameter, circularity, decentration, and package. We found that there was a positive correlation between the area of capsulorrhexis and the deviation from predicted refraction at 1 month and 3 months in the Tecnis IOL group, as well as horizontal diameter at 1 month and vertical diameter at 3 months. For every 1-mm^2^ increase in size, the deviation from predicted refraction will increase by 0.0152 D. For every 1-mm increase in horizontal diameter, the deviation from predicted refraction will increase by 0.07 D. In addition, for every 1-mm increase in vertical diameter, the deviation from predicted refraction will increase by 0.05 D. For the 509 M IOL, there were no significant correlations between the morphological parameters and the deviation from predicted refraction (all *P* > 0.05). This single-piece hydrophilic acrylic intraocular lens has good flexibility and the loop can adapt to different sizes of the capsule bag due to the unique design of the bending loops so that asymmetric contraction force in different directions can be balanced and maintain stability after IOL implantation. Yu Fang et al. [16] compared the stability of a single-piece and a three-piece aspheric intraocular lens from the same company and found that the stability of the single-piece IOL is better than that of the three-piece IOL.

Simultaneously, in the correlation analysis between the capsulorrhexis morphological features and the ELP, it was found that the area, horizontal diameter, and vertical diameter of the capsulorrhexis were correlated with the ELP in the Tecnis IOL group, while the package was correlated with ELP in both groups. Studies have shown that in all capsulorrhexis parameters, the package affects the horizontal and vertical shift of the intraocular lens. Improvements to the package of capsulorrhexis leads to a smaller shift of the intraocular lens, a more stable IOL position, a central positon and a smaller refractive deviation after surgery [17]. Dick et al. proposed that the centre of the capsulorrhexis should be located on the optic axis, that the capsulorrhexis should be perfectly round and that the best diameter is 5.25 mm, which could be the most effective way to prevent the aberration, coma, and ametropia caused by the contraction of the capsular bag, the posterior cataract, and the tilt of the intraocular lens [18]. Kim et al. [19] proposed that the posterior continuous circular capsulorrhexis has a more-stable refractive outcome and prevents the effect of posterior capsular opacity on the stability of the IOL. In recent years, the emergence of electronic capsulorrhexis and femtosecond-assisted capsulorrhexis has enabled the capsulorrhexis to develop in a more precise direction. It was found that the excellent rate of the conventional artificial capsulorrhexis group was only 20%, the failure rate was 60%,while the excellent rate of the electronic capsulorrhexis group was 100%, and the difference between the two groups was statistically significant [20].

In summary, the morphological factors of the capsulorrhexis can affect ELP value, ultimately affecting the postoperative diopter. At the time of implantation of C-loop haptic IOLs, the surgeon should pay attention to attention the size of the capsulorrhexis and the package. These factors have influence on ELP and postoperative diopter. For plate haptic IOLs, the requirements for the capsulorrhexis can be slightly fewer, the package of the capsulorrhexis is the most important factor for the intraocular lens, which can make the position of the intraocular lens more stable postoperatively. The four-point force is symmetrical, which is effective at reducing and offsetting each other. The buffering capacity prevents it from being affected by external forces, and reduces the tilt and decentration of the IOL.

However, the effective position of the intraocular lens is affected not only by the capsulorrhexis, but also by the tension of capsular, the supporting force and the lens zonules. The intraocular lens positions the visual axis under the combined action of these three forces. The 509 M IOL group showed an increase in ELP at 1 week and 1 month after surgery, We suspected that after the lens capsule lost its original support, the lens zonules were relaxed by the support of the intraocular lens. The postoperative intraocular lens position moves backwards, which increases the ELP, but this change in ELP has little effect on the change of the postoperative diopter. We suspected that as the ELP increases, the interactions among hydrophilic material IOL, ciliary muscle, lens zonules and capsules can change the postoperative diopter and maintain the stability of the diopter. In the Tecnis IOL group, the ELP was relatively stable, and there is no significant change in postoperative diopter, but if ELP changes, it affects postoperative diopter. We consider that this hydrophobic acrylate IOL can increase adhesion to capsules. Compared to 509 M IOLs, Tecnis IOLs may be more closely connected to the capsule bag. Although the intraocular lens is relatively stable, once the intraocular force is given, the ELP will increase and affect postoperative diopter, the intraocular lens may not have enough flexibility to keep the diopter stable as that of hydrophilic IOLs. Therefore, each type of intraocular lens has advantages and disadvantages, and various factors must be considered in the selection of IOLs.

In the future, we will increase the sample size, increase the different types of intraocular lenses, and extend the follow-up time to supplement and improve these data and provide a reference and clinical basis for the size and shape of the capsulorrhexis for different intraocular lenses.

## Conclusions

In summary, the current study compared the postoperative refractive outcomes and the effective position of 509 M IOLs and Tecnis IOLs. For Tecnis IOLs, the size of the capsulorrhexis and the package are important factors influencing the ELP and the postoperative refractive outcomes, while for 509 M IOLs, the package is the only factor of capsulorrhexis influencing the ELP. Only the full mastery of CCC can minimize the postoperative refractive shift and the ELP, which will enable the patients to achieve satisfactory visual effects after surgery.

## Abbreviations

ANOVA: Analysis of Variance
CCC: Continuous circular capsulorrhexis
D: Diopters
ELP: Effective position of the intraocular lens
IOL: Intraocular lens
SE: Spherical equivalent
